# Distinct mechanism of cervical cancer cell death caused by the investigational new drug SHetA2

**DOI:** 10.3389/fonc.2022.958536

**Published:** 2022-09-20

**Authors:** Rajani Rai, Vishal Chandra, Amy L. Kennedy, Rosemary E. Zuna, Doris Mangiaracina Benbrook

**Affiliations:** ^1^ Gynecologic Oncology, Stephenson Cancer Center, University of Oklahoma Health Sciences Center, Oklahoma, OK, United States; ^2^ Department of Pathology, University of Oklahoma Health Sciences Center, Oklahoma, OK, United States

**Keywords:** cervical cancer, SHetA2, mitochondria, mitophagy, apoptosis inducing factor, heat shock cognate 70, cell death, heat shock protein

## Abstract

Drug-targetable vulnerabilities of cancer cells include their dependence on heat shock proteins (HSPs) to support elevated mitochondrial metabolism and counteract cell death factors. The investigational new drug SHetA2 targets these vulnerabilities in ovarian and endometrial cancer cells by disrupting complexes of the mortalin HSP with its client proteins (mitochondrial support proteins, metabolic enzymes, p53) leading to mitochondrial leakage of cytochrome c and apoptosis-inducing factor (AIF), and caspase-dependent apoptosis. Our objective was to evaluate the roles of mitochondrial damage and another SHetA2-target HSP protein, cytoplasmic heat shock cognate 70 (hsc70), in the mechanism of SHetA2 killing of cervical cancer cells. Cervical cancer cells responded to SHetA2 with excessive mitophagy that did not deter AIF leakage into the cytoplasm. Then, hsc70 was unable to prevent cytoplasmic AIF nuclear translocation and promotion of DNA damage and cell death, because SHetA2 disrupted hsc70/AIF complexes. The Cancer Genome Atlas analysis found that overexpression of hsc70, but not mortalin, was associated with worse cervical cancer patient survival. Use of specific inhibitors documented that AIF and mitophagy, but not caspases, contributed to the mechanism of SHetA2-induced cell death in cervical cancer cells. As validation, excessive mitophagy and lack of caspase activation were observed in SHetA2-inhibited xenograft tumors.

## Introduction

Globally, cervical cancer occurred in 570,000 women and caused 311,000 deaths in 2018 (1). This cancer is diagnosed primarily in middle-aged women in the age range where women commonly have productive careers and dependent families. The vast majority of cervical cancers are caused by high risk Human Papillomavirus (hrHPV) and subsequent genomic instability. HPV vaccines have reduced cervical cancer incidence, especially in developed countries, however further reduction is not likely to be seen in the next decade due to low vaccination rates, emersion of rare oncogenic HPV variants and lack of effect of vaccines on pre-existing hrHPV infections. The standard of care therapy for cervical cancer is based on combinations of radiation and chemotherapy. These modalities are highly toxic and cause significant morbidity (2, 3). Although recent FDA approvals have added immune-based therapy to this armament, cervical cancer lags behind most other cancers in having molecular-based drugs available for therapeutic use.

Heat shock proteins (HSPs) are rational targets for development of new cancer therapeutics because cancer cells become dependent upon increased levels of HSPs to maintain their unstably-elevated proliferative and metabolic states (4). HSPs are categorized based on their molecular weights. The 70 Kd family of HSPs (HSP70s) consists of 14 members, which each have unique and redundant functions throughout the cell (5). The mortalin member of this family is localized throughout the cells, but it’s primary location and function are in maintaining mitochondrial health (5, 6). Increased levels of mortalin promote carcinogenesis and cancer progression by supporting overactive mitochondria and sequestering p53 in the cytoplasm away from the mitochondria and nucleus where it can initiate apoptosis (7–10). Mortalin has also been shown to support carcinogenesis and cancer cells driven by rearranged during transfection (RET) proto-oncogene, mutant Ras and Raf oncoproteins and MEK-ERK signaling activity (11–13). These cancer supporting activities include regulation of mitochondrial bioenergetics (13) and the mitochondrial membrane potential transition pore (14, 15). The investigational new drug, SHetA2, interferes with mortalin support of cancer cells by disrupting mortalin complexes with client proteins (16). Client proteins shown to be blocked from binding mortalin by SHetA2 in endometrial cancer cells include proteins involved in mitochondrial metabolism and calcium import (17). The mitochondrial damage eventually leads to mitochondrial release of cytochrome c, which activates caspases to cause apoptotic cell death, and apoptosis-inducing factor (AIF), which translocates to the nucleus and promotes DNA damage and cell death (18). An important mortalin client protein is p53, which when released from mortalin by SHetA2 translocates to the mitochondria and nucleus where it induces apoptosis (8).

While the role of mortalin has been well established in the mechanism of SHetA2-induced ovarian and endometrial cancer cell death, the involvement of another SHetA2-binding HSP70 protein called heat shock cognate 70 (hsc70) (16) has not yet been studied. The hsc70 family member has the potential to interfere with the AIF-mediated mechanism of SHetA2-induced cell death because it sequesters AIF in cytoplasm away from the nucleus (19). It is likely that the hrHPV-driven intracellular environment of cervical cancer cells, which includes decreased p53, cause them to respond differently to SHetA2 compared to cells of other gynecologic cancers. A previous study demonstrated the cell cycle regulatory mechanism of SHetA2 and its synergistic interaction with cyclin dependent kinase 4/6 inhibitors (20), however no studies have yet been done on the mechanism of SHetA2-induced cell death of cervical cancer cells.

The objective of this study was to determine how cervical cancer cells respond to, and eventually die, from SHetA2 treatment. The known deleterious effects of SHetA2 on mitochondria in other cancers were evaluated in more depth in cervical cancer cells and tumors. The hsc70 chaperone target of SHetA2 was evaluated as a potential target for cervical cancer treatment and for its role in the mechanism of SHetA2 killing of cervical cancer cells. Specific molecular and cellular responses to SHetA2 were tested for their roles as mediators of survival versus death.

## Results

### SHetA2 inhibits mitochondrial function and biogenesis in cervical cancer cells

**Figure 1:**
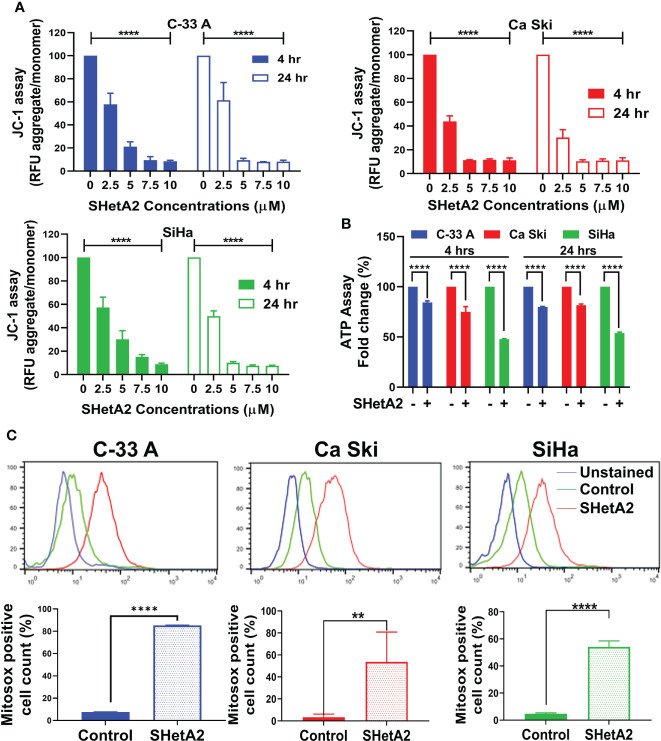
SHetA2 induces mitochondrial depolarization and loss of ATP, **(A)**: Relative fluorescence (RFU) of JC-1 dye, and ratios of JC-1 aggregate to monomer expressed as mean ± SD and analyzed using two-way ANOVA. **(B)**, ATP levels in cells treated with SHetA2 (10 µM) or vehicle analyzed using *t*-tests. **(C)**, Representative histograms from Flow cytometric analysis of MitoSOX staining of cells treated with SHetA2 (10 µM) or vehicle for 24 hours (left) and average (mean ± SD) data of three independent experiments were analyzed by t-tests (right). **p ≤ 0.01, ****p ≤ 0.0001 when compared with respective control.

**Figure 2:**
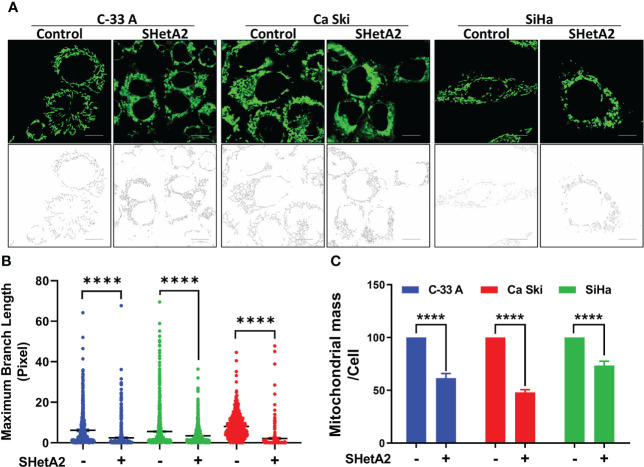
SHetA2 reduces mitochondrial networking and mass: **(A, B)**, Confocal imaging of MitoTracker™ Green staining of cells treated with 10 µM SHetA2 (**A**, upper panel). Representative imaging of mitochondrial networks analyzed using Image J software (**A**, lower panel). Maximum mitochondrial branch lengths were compared by *t*-tests **(B)**. **(C)**, C-33 A, Ca Ski and SiHa cervical cancer cells treated with 10 µM SHetA2 or vehicle for 24 hours were stained with MitoTracker™ Green and Hoechst. The fluorescence was imaged and analyzed by using the Operetta^®^High Content Imaging System. Mitochondrial mass in cells treated with SHetA2 or vehicle for 24 hours were measured by ratios of MitoTracker™ Green to Hoechst staining, presented as mean ± SD and analyzed using *t*-tests. ****p ≤ 0.0001 when compared with respective control.

**Figure 3:**
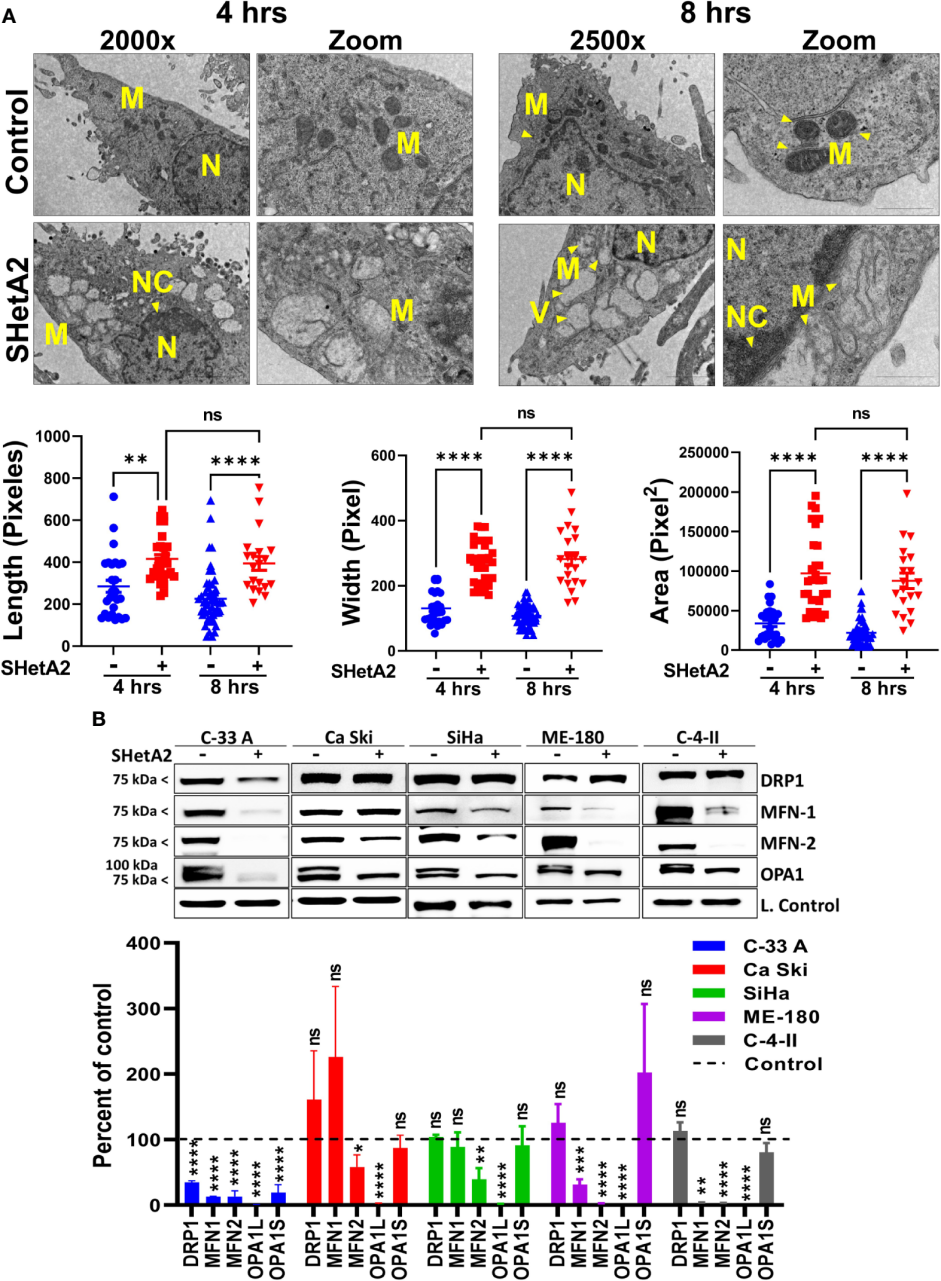
SHetA2 alters mitochondrial morphology and downregulate mitochondrial dynamics proteins: **(A)**, TEM of SiHa cells treated with SHetA2 (10 µM) or vehicle for 4 and 8 hours. Representative images showing mitochondria (M), nucleus (N), nuclear condensation (NC) and autophagic vesicles (V) are shown (**A**, upper panel). For the quantification of mitochondrial size, mitochondria with *cristae* structure were measured for length, width and total area and compared by *t*-tests (**A,** lower panel). **(B)**, Western blots of cells treated with DMSO or 10 µM SHetA2 for 48 hours (upper panel). GAPDH (for C-33 A, Ca Ski, SiHa, and C-4-II), or cyclophilin B (for SiHa**)** or α-tubulin (for ME-180) were used as loading controls (L. Control). Densitometric analysis of the bands are shown as mean ± SD and were compared using a *t*-test (lower panel). *p ≤ 0.05, **p ≤ 0.01, ***p ≤ 0.001, ****p ≤ 0.0001 NS-not significant when compared with respective control.

Based on the essential roles of mortalin in the import of nuclear-encoded mitochondrial proteins and maintenance of mitochondrial function (9), and the SHetA2 disruption of mortalin complexes in ovarian cancer cells (4, 5), we predicted that SHetA2 damages mitochondria in cervical cancer cells. In this study, SHetA2 effects on mitochondria were evaluated in three human cervical cancer cell lines C-33 A, Ca Ski and SiHa after 4 and 24 hours of treatment. As expected, SHetA2 reduced mitochondrial membrane potential (MMP) in all cell lines in a dose- and time-dependent manner (**Figure 1A**). SHetA2 also reduced total cellular ATP to similar levels at 4 and 24 hours in all three cell lines indicating decreased mitochondrial function (**Figure 1B**). To assess mitochondrial dysfunction, flow cytometric analysis of Mitosox was used to measure mitochondrial ROS. The results showed that SHetA2 increased mitochondrial ROS in all three cell lines, as well as the ME-180 and C-4-II human cervical cancer cell lines (**Figure 1C**; **Supplemental Figures 1A, B**). SHetA2 effects observed in all three cell lines using Mitotracker staining included reduction of mitochondrial networking (**Figures 2A, B**) and mitochondrial mass as measured by the ratio of Mitotracker to Hoechst nuclear staining (**Figure 2C**; **Supplemental Figure 1C**). To better visualize SHetA2 effects on mitochondria, we used transmission electron microscopy (TEM) of SiHa cultures treated with 10 µM SHetA2 or vehicle for 4 hours or 8 hours (**Figure 3A** upper panel). At both treatment times, SHetA2-treated cells had fewer mitochondria, which were swollen (significantly greater length, width, and total area) (**Figure 3A**), and increased autophagic vesicles and chromatin condensation, in comparison to controls (**Figure 3A**).

To evaluate the molecular mechanism of these mitochondrial events, proteins were extracted from the cells and evaluated by western blots. SHetA2 reduced mitochondrial proteins involved in fusion (mitofusin 1/MFN1, mitofusin 2/MFN2, and long-form of outcome predictor in acute leukemia 1/OPA1L), but not those involved in fission (dynamin-related protein 1/Drp1) in the five cell lines tested (**Figure 3B**). Exceptions were significant reduction of Drp1 in C-33 A, and lack of significant MFN-1 reductions in Ca Ski and SiHa. The OPA1S isoform, which is dispensable for fission under stressed conditions (21), was not reduced in any of the cell lines except for C-33 A (**Figure 3B**). Taken together, these results demonstrate that mitochondrial biogenesis, fusion, and function are disrupted by SHetA2 in cervical cancer cells.

### Cells respond to SHetA2-induced mitochondrial damage with mitophagy that contributes to the mechanism of cell death

**Figure 4:**
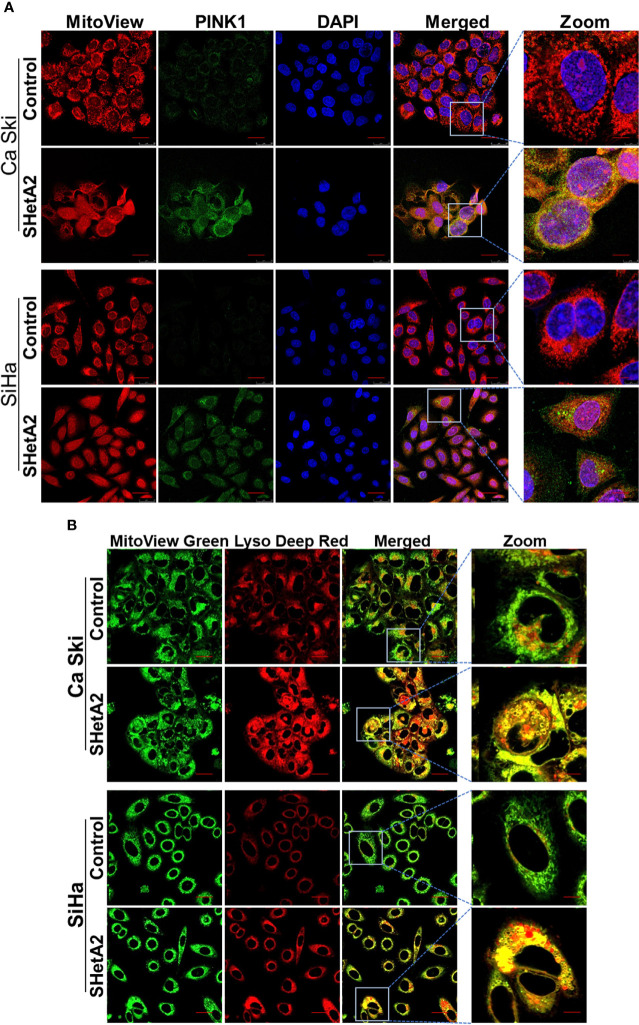
SHetA2 induces mitophagy in cervical cancer cells: **(A, B)**, Mitophagy induction was demonstrated by confocal imaging of cervical cancer cells treated with SHetA2 (10 µM) for 24 hours and stained with Pink1 (green) mitotracker red (red) and DAPI (blue) **(A)**, or with MitoView green (green) and Lyso Deep Red (red) dye **(B)**.

Based on the observed mitochondrial damage, we predicted that the cervical cancer cells respond by elevating mitochondria-selective autophagy (mitophagy) (22) to eliminate the damaged mitochondria and recycle the components. In concordance with induction of mitophagy in SHetA2-treated cells, confocal imaging demonstrated increased expression of the mitophagy marker Pink1 and its co-localization with the mitochondrial marker (mitotracker red, **Figure 4A** and **Supplementary Figure 2**), in addition to colocalization of lysosome dye with mitotracker (**Figure 4B**).

**Figure 5:**
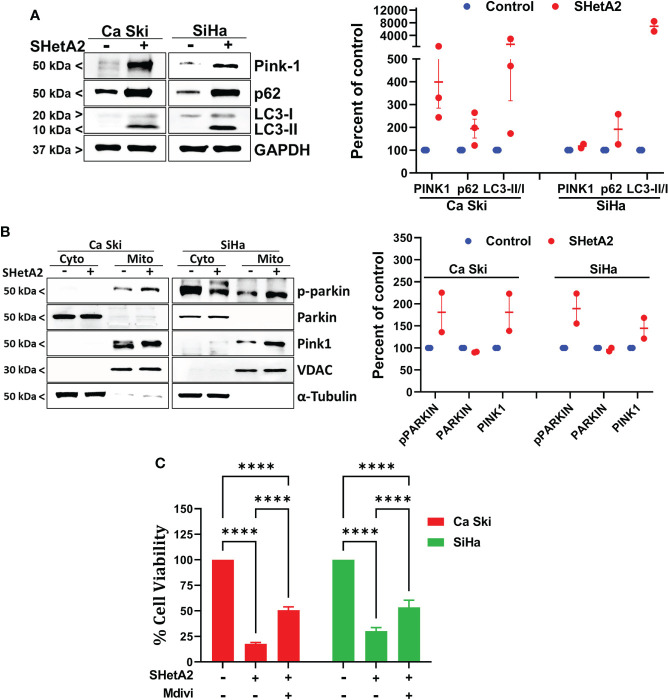
SHetA2 induced mitophagy contribute to cervical cancer cell death: **(A)**, Western blot analysis of Ca Ski and SiHa cell treated with SHetA2 (10 µM) for 48 hours (left panel). Densitometric analysis of the bands (Pink-1, p62 and LC3-II/LC3-I ratio) are shown as mean ± SD and were compared using a *t*-test (right panel). **(B)**, Western blot analysis of cytoplasmic and mitochondrial fractions of Ca Ski and SiHa cell treated with SHetA2 (10 µM) for 48 hours (left panel). Densitometric analysis of the bands are shown as mean ± SD and were compared using a *t*-test (right panel). **(C)**, Cervical cancer cells were pre-treated with 10 µM Mdivi-1 for 4 hours followed by SHetA2 (10 µM) for 72 hours and MTT assay was performed. ****p ≤ 0.0001 when compared with respective control.

To confirm at the molecular level that SHetA2 caused mitophagy, western blots of SHetA2-treated cultures were stained for various molecular markers of autophagy (microtubule-associated protein 1A/1B-light chain 3 (LC3)-II to the LC3-I protein modification) and mitophagy (Pink-1 and p62). In both Ca Ski and SiHa cells, SHetA2 induced increased levels Pink-1, p62, and LC3-II/LC3-I ratios (**Figure 5A**). For further validation of mitophagy, cytoplasmic- and mitochondria-enriched fractions of the cell extracts were collected and evaluated by western blot. In both Ca Ski and SiHa cells, SHetA2 caused recruitment of Pink1 and phosho-parkin (p-parkin, activated parkin) to mitochondria (**Figure 5B**).

Since mitophagy can serve as a cell survival function by eliminating and recycling damaged mitochondria, or contribute to the cell death by excessive depletion of mitochondria, we next tested if inhibition of mitophagy increased or decreased SHetA2 sensitivities of Ca Ski and SiHa cell lines using Mdivi-1, a selective cell-permeable inhibitor of mitochondrial division DRP1 (dynamin-related GTPase) and mitochondrial division Dynamin I (Dnm1). Inhibition of mitophagy by Mdivi-1 was found to partially, but significantly, reduce SHetA2 mediated cell toxicity (**Figure 5C**).

### Ultimately, SHetA2 treatment results in AIF-induced, caspase-independent apoptosis

**Figure 6:**
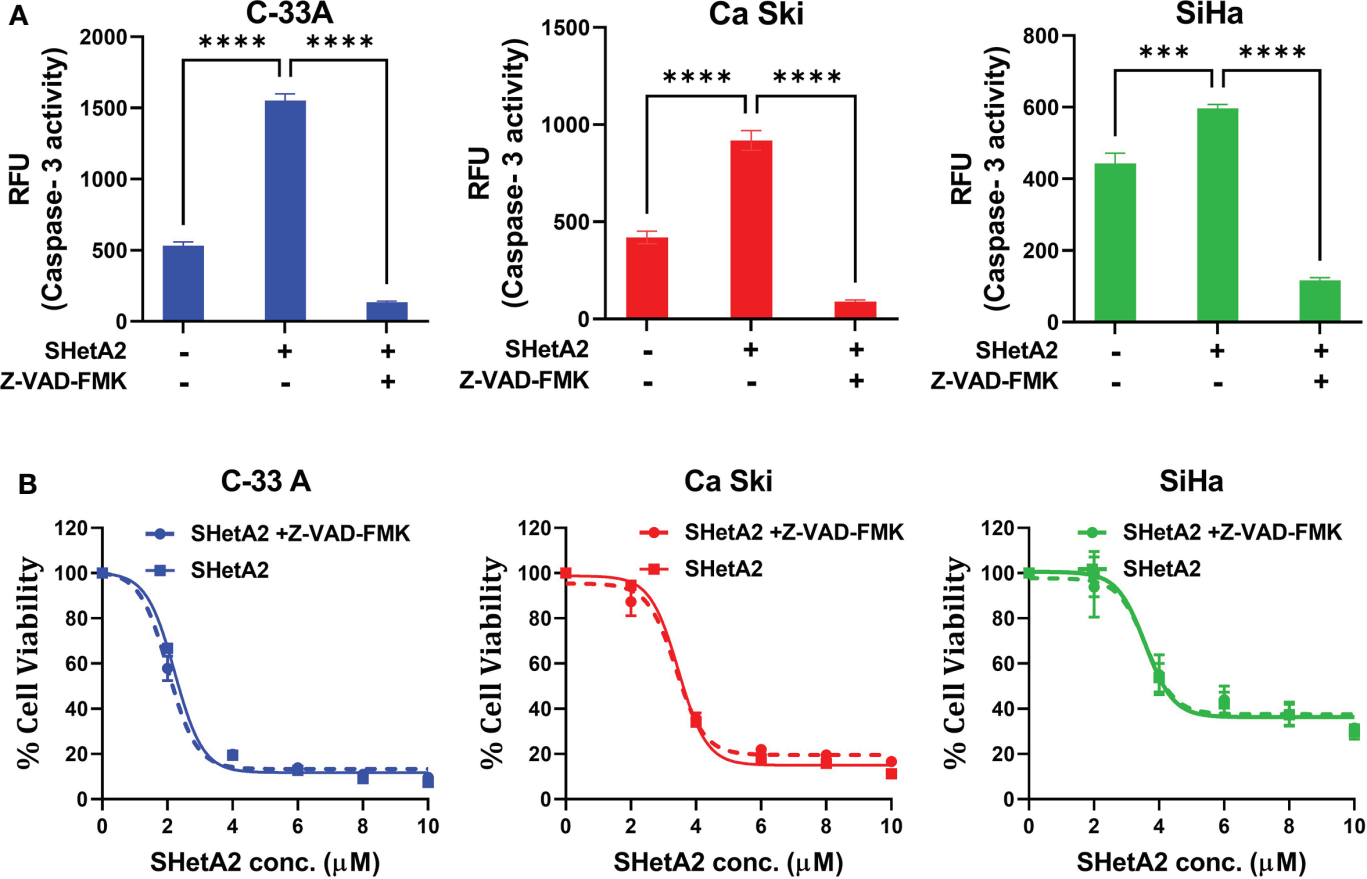
SHetA2 inhibits metabolic viability of cervical cancer cell in caspase-independent manner: **(A)**, Caspase 3 activity in cervical cancer cells treated for 48 hours with vehicle, 10 µM SHetA2, or a combination of pan-caspase inhibitor (Z-VAD-FMK, 30 µM, pre-treatment for 3 hours) and SHetA2. A one-way ANOVA was used for statistical analysis. **(B)**, Representative dose response curves of C-33 A, Ca Ski and SiHa cells pretreated with 30 µM of pan-caspase inhibitor (Z-VAD-FMK) for 3 hours followed by SHetA2 treatment at different doses for 72 hours. ***p ≤ 0.001, ****p ≤ 0.0001 when compared with respective control.

We next determined the mechanism of SHetA2-induced cervical cancer cell death. SHetA2 induced caspase-dependent apoptosis in ovarian, kidney, and lung cancer cells (23–26). As expected and consistent with our previous SiHa xenograft study (6), SHetA2 significantly induced caspase-3 activity in cervical cancer cell lines (**Figure 6A**). Inhibition of caspase activity with a pan-caspase inhibitor, as confirmed by caspase-3 assay (**Figure 6A**), did not significantly alter SHetA2 cytotoxicity, suggesting that SHetA2 works independently of caspase activity in cervical cancer cell lines (**Figure 6B**).

**Figure 7:**
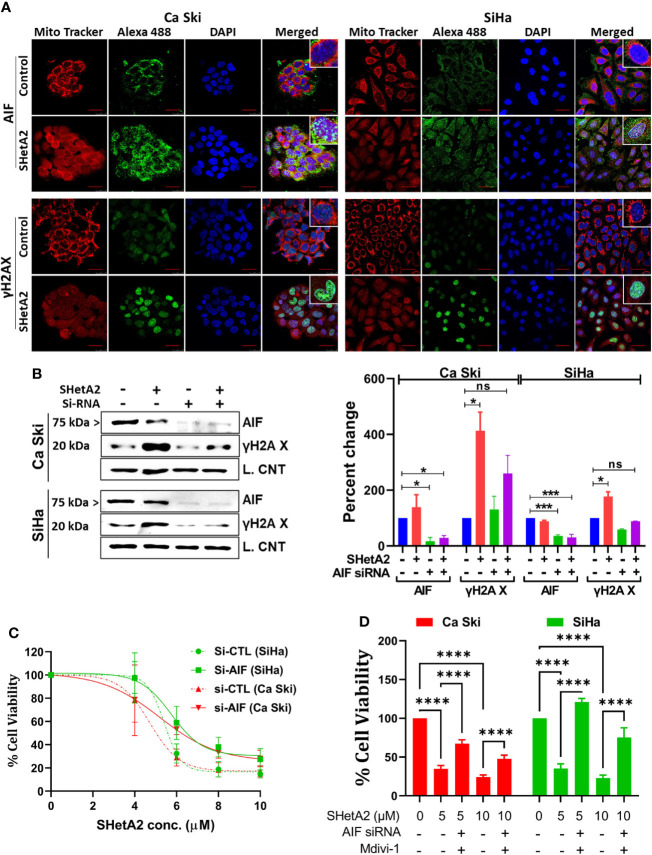
SHetA2 induces AIF mediated DNA damage contributing cell death: **(A)**, Confocal microscopy of cells stained with anti -AIF (green) or -γH2AX (green), mitotracker (red) and DAPI (blue) after treatment with 10 µM SHetA2 for 24 hours. **(B)**, Western blots of cells transfected with either non-target si-RNA (si-CTL) or si-AIF and treated with SHetA2 (10 µM) (left panel) and analyzed by densitometry analysis (right panel). GAPDH (Ca Ski, SiHa), or cyclophilin B (SiHa**)** were used as loading controls (L. Control). **(C)**, MTT assays of cells transfected with either si-CTL or si-AIF. **(D)**, cervical cancer cells were transfected with either si-CTL or si-AIF. Following pretreatment with Mdivi-1, transfected cells were treated with SHetA2 at indicated concentration for 72 hours and MTT assay was performed. *p ≤ 0.05, ***p ≤ 0.001, ****p ≤ 0.0001 when compared with respective control.

To evaluate a potential alternate form of mitochondrial-mediated death, SHetA2 effects on AIF localization and DNA damage were evaluated. Immunofluorescence confocal imaging documented that SHetA2 significantly increased AIF nuclear localization and nuclear staining of γH2AX, as an indicator of DNA damage (**Figure 7A**). Electron microscopy images confirmed nuclear condensation in SHetA2-treated SiHa cells (**Figure 3A**). siRNA reduction of AIF, confirmed by western blot (**Figure 7B**), prevented SHetA2 induction of γH2AX (**Figure 7B**). Metabolic viability assays confirmed reduction of SHetA2 potency and efficacy by AIF siRNA in Ca Ski and SiHa (**Figure 7C**). Further, combined inhibition of both mitophagy by Mdivi-1 and AIF by siRNA was found to significantly counteract SHetA2-mediated cell death in cervical cancer (**Figure 7D**). These results document that SHetA2 treatment ultimately results in cell death through mitophagy and AIF migration from mitochondria to the nucleus where it causes DNA damage. In contrast to other cancer types, caspase activation is not required for the mechanism of SHetA2-induced death in cervical cancer.

### Role of hsc70 in cervical cancer and the SHetA2 cell death mechanism

**Figure 8:**
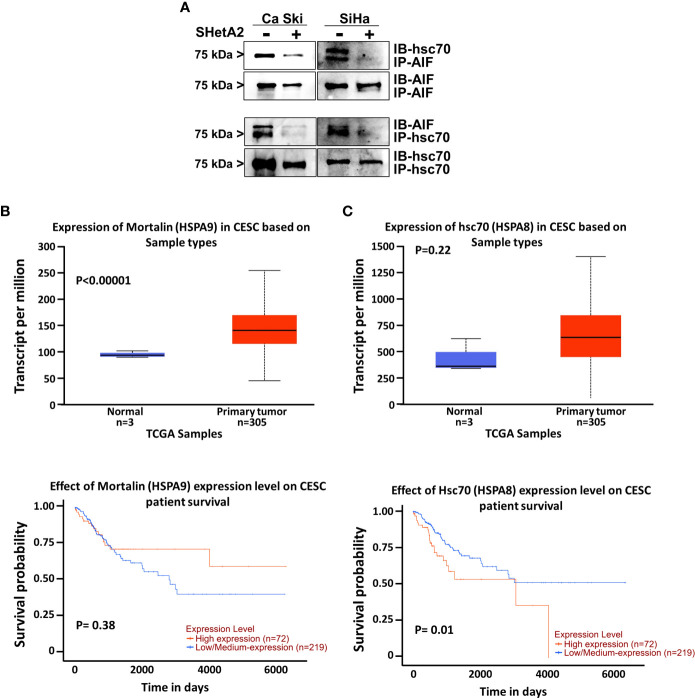
Role of hsc70 in cervical cancer and SHetA2 mediated cell death mechanism: **(A)**, Co-immunoprecipitation assays of cervical cancer cells treated with 10 µM SHetA2 for 24 hours. **(B, C)**, UALCAN analysis of TCGA data for expression of mountain (HSPA9, **B**) and hsc70 (HSPA8, **C** upper panel) mRNA in cervical squamous cell carcinoma (CESC) compared to healthy tissue and comparison of their high versus low expression with cervical squamous cell cancer patient survival probability (lower panel **B, C**).

Because hsc70 was identified as an SHetA2 binding protein (16) and can prevent AIF nuclear localization (18), we predicted that SHetA2 disrupts hsc70 binding to AIF. To test this possibility, we immunoprecipitated proteins from Ca Ski and SiHa protein extracts using hsc70 or AIF antibodies and probed the precipitates for the predicted binding proteins. Western blots of the precipitates confirmed that hsc70 co-immunoprecipitated with AIF and that AIF co-immunoprecipitated with hsc70, while SHetA2 treatment of cells for 24-hours, prevented these co-immunoprecipitations (**Figure 8A**). Taken together, these results demonstrate that hsc70 may interfere with SHetA2-induced nuclear AIF localization and DNA damage.

To evaluate the relevance of the SHetA2 hsc70 and mortalin targets in cervical cancer, we probed The Cancer Genome Atlas (TCGA) data using a public website (http://ualcan.path.uab.edu/index.html). This analysis demonstrated significant mortalin overexpression in cervical squamous cell carcinoma compared to healthy tissue (p <0.0001), but this was not associated with patient survival (**Figures 8B**). Although hsc70 elevation in cervical squamous cell carcinoma was not statistically significant, there was a significant association of high hsc70 expression with reduced survival probability of patients (p = 0.01, **Figures 8C**).

### SHetA2 induces mitophagy in, and inhibits growth of, Ca Ski Xenograft Tumors

**Figure 9:**
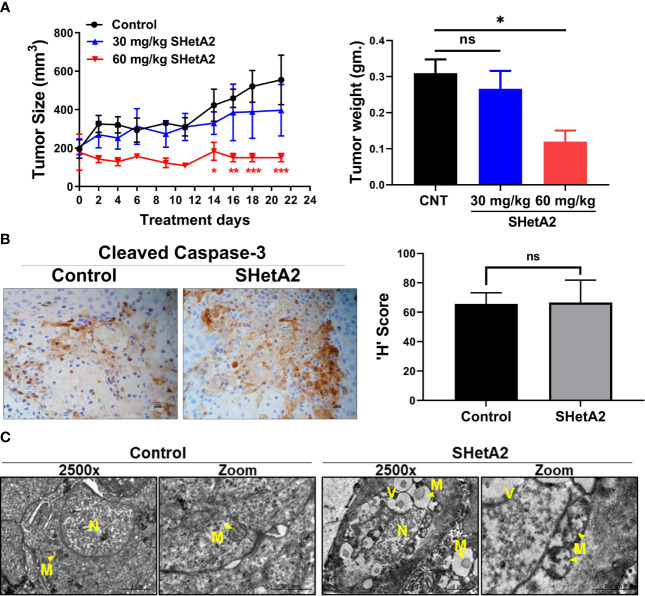
SHetA2 induces mitophagy and inhibits growth of cervical cancer tumor *in-vivo*: **(A)**, Average tumor volumes of Ca Ski xenograft in mice treated with orally SHetA2 (30 and 60 mg/kg) or vehicle for 21 days. The average tumor volume during the treatment period (left panel) and tumor weight (right panel) were compared by two-way and one-way ANOVA, respectively. **(B)**, expression of cleaved caspase-3 was measured on the xenograft tumor tissue treated with SHetA2 (60mg/kg group) or control by Immunohistochemistry and the representative images are shown (left panel). The H score was compared between both groups by using Student’s *t*-tests (right panel). **(C)**, TEM images of Ca Ski xenograft tumors control or 30 mg/kg/day SHetA2 treatment groups. *p ≤ 0.05, **p ≤ 0.01, ***p ≤ 0.001, ns; not significant when compared with respective control.

We next determined if these mitochondrial effects are also caused by SHetA2 treatment in an animal model. Previously, we demonstrated that oral administration of 60 mg/kg SHetA2 significantly inhibited the growth of SiHa cervical cancer xenografts without inducing toxicity (6). In this study, two oral doses (30 and 60 mg/kg/day) of SHetA2 induced a dose-responsive reduction in Ca Ski xenograft tumor growth (**Figure 9A**). The 60 mg/kg/day dose was statistically significant for reduced tumor volume (ANOVA: p = 0.0360) and tumor weight (ANOVA: p = 0.0266) compared to controls. Consistent with the caspase-independent nature of SHetA2 cell killing in cervical cancer cell line cultures, SHetA2 did not significantly alter cleaved caspase 3 expression in Ca Ski xenografts (**Figure 9B**). TEM of tumors from the 30 mg/kg SHetA2 treated Ca Ski xenografts revealed swollen mitochondria and accumulation of autophagic vesicles (**Figure 9C**). The majority of autophagic vesicles harbored mitochondria at various stages of degradation. This observation indicates that SHetA2 induced mitochondria-selective autophagy (mitophagy) in association with inhibition of cervical cancer tumor growth.

## Discussion

**Figure 10:**
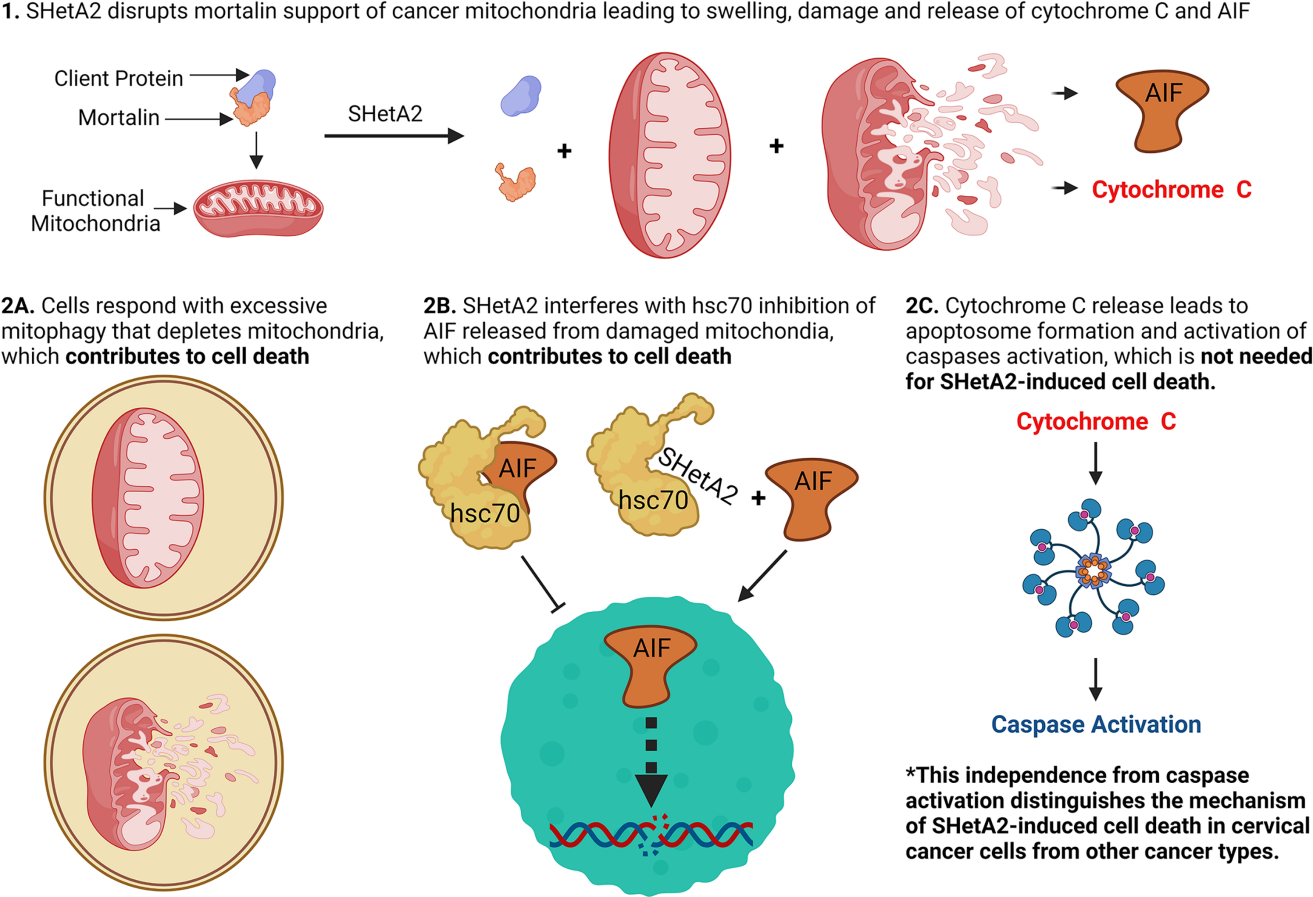
Model of SHetA2 mechanism of causing cervical cancer cell death.

The results of this study demonstrate that SHetA2 kills cervical cancer cells through a similar but, distinct mechanism to that demonstrated in other cancer types. Similar to observations in other cancer types, SHetA2 caused mitochondrial damage which led to activation of caspases and AIF translocation to the nucleus (17, 23–26). In contrast to the dependence on caspase activity for SHetA2 cell killing of ovarian, kidney, and lung cancer cells (21–24), the mechanism in cervical cancer cells occurred independently of caspase activation as documented by a lack of caspase inhibitor effect on SHetA2 cytotoxicity. Because cervical cancer cells are capable of undergoing caspase-dependent apoptosis (27, 28), the caspase activation we observed in SHetA2-treated cells could be contributing, but not required, for SHetA2-induced cell death, because the SHetA2-induced mitophagy and nuclear relocation of AIF could be compensating for the inhibition of caspase activity (**Figure 10**).

The observation of SHetA2 induction of mitophagy had not been evaluated in previous studies. The SHetA2-induced mitophagy was observed in both cell culture and xenograft tumors. In cell culture, the mitophagy was verified through upregulation of autophagy markers and co-localization of mitophagy-specific proteins with mitochondria and lysosomes. In the xenograft tumors, the levels of mitophagy were excessive with the vast majority of autophagic vesicles observed to contain single mitochondria. Thus, it was not surprising to observe that inhibition of mitophagy decreased the SHetA2 cytotoxicity. The reason for the mitophagy contributing to the mechanism of cell death instead of serving in a survival role is likely due to the excessive nature of the mitophagy eliminating too many mitochondria before more can be made. Combined inhibition of mitophagy and AIF significantly reduced, but did not completely eliminate SHetA2 cytotoxicity. Reasons for the lack of complete prevention could be that the doses and treatment times were not optimized and/or that other factors also contribute to the mechanism of cell death.

The lack of caspase dependency of SHetA2-induced cell death in cervical cancer cells could be due to higher capacity of cancer cells to induce mitophagy or higher dependency on hsc70. Currently, it is not known if mitophagy or hsc70 play causative roles in the reason why caspases are not required for SHetA2-induced cell death in cervical cancer cells while they are required in other cancer types. The mitophagy capacity of cervical cancer compared to other cancer types has not been reported. TCGA data probed with the ualcan.path.uab.edu website shows that cervical cancer does not express higher levels of *HSPA8* (gene encoding hsc70) mRNA in comparison to other cancer types. Cervical cancer is one of the 5 out of 32 cancers evaluated by TCGA that have significant associations of high *HSPA8* mRNA expression with worse survival. The others are invasive breast cancer, kidney renal clear cell carcinoma, liver hepatocellular carcinoma and mesothelioma. Thus, it is possible that cervical carcinogenesis involves development of dependency on hsc70 protein expression and that hsc70 contributes to the different response of cervical cancer cells to SHetA2 in comparison to other cancers. Studies of hsc70 are technically complicated by the 85% homology between hsc70 and HSP70 and cross-reactivity of antibodies to these proteins. (16)

In preclinical studies, SHetA2 was shown to act synergistically with a p53 reactivator in ovarian cancer (8) and with a CDK4/6 inhibitor in cervical cancer (20). These drug combination studies were pursued based on knowledge of the mechanism of SHetA2 as a single agent in cancer cells. The observation in this study that the SHetA2 cell killing mechanism is distinct in cervical cancer cells indicates that the efficacies of drug combinations may differ depending on the cancer type. Knowledge of drug toxicities are also important to take into consideration in designing drug combination trials. One-month long toxicity studies in rats and dogs documented that SHetA2 does not cause toxicity at doses 50 fold higher than doses which reduced tumor growth in rodent models (29). The potential for SHetA2 to be used in prevention studies is supported by its lack of mutagenicity, carcinogenicity, and skin irritancy (30–32). The preclinical SHetA2 drug combination studies conducted to-date observed no significant toxicities in any of the drug treatment groups (8, 20).

## Future directions

Currently, an oral capsule formulation of SHetA2 is being evaluated in a first-in-human Phase 1 clinical trial of advanced and recurrent, ovarian, cervical and endometrial cancers (clinicaltrials.gov: NCT04928508). Pending determination of a safe recommended phase 2 dose in this trial, SHetA2 combination studies can be pursued. The caspase-independence of the SHetA2 mechanism observed in this study, suggest that SHetA2 combinations with drugs that act *via* caspases may be worth evaluating, while drugs that inhibit mitophagy or upregulate hsc70 would not be good choices for SHetA2 drug combination studies.

The minimal-to-no toxicity observed for SHetA2 to date support further development of SHetA2 as a chemoprevention drug and food additive. Women diagnosed with cervical intraepithelial neoplasia 3 (CIN3) have significant risk of developing cervical cancer and the standard of care for these patients is to have their cervical lesions removed by a loop electrosurgical excision procedure (LEEP), large loop excision of the transformation zone (LLETZ) or cold-knife conization (33). Depending on the Country, women diagnosed with CIN 2 are triaged to either active surveillance or one of these surgical procedures (34). Many women who are triaged to active surveillance feel anxiety about their risk of developing cervical cancer (35). The LEEP/LLETZ/conization procedures are only partially effective as there remains significant risk for future development of neoplasia (36) and cancer (37), especially in hrHPV positive women (38). Furthermore, these surgical procedures cause increased risk of worse obstetrics and neonatal outcomes for those pursuing reproduction after the procedure (39). This situation provides an opportunity to apply SHetA2 as a means to prevent progression of CIN to cancer. Cancer prevention studies of SHetA2 are justified by preclinical studies that documented the ability of SHetA2 to prevent development of the abnormal cancerous phenotype in organotypic cultures of endometrial epithelial cells (40) and in an animal model of colorectal cancer (41). A vaginal suppository formulation of SHetA2 has been developed, which could apply drug directly to the CIN lesion and avoid potential systemic side effects (42–45).

SHetA2 has been shown to bind three related HSP70 proteins, mortalin, Grp78 and hsc70 (16). While the role of mortalin in the mechanism of SHetA2 has been extensively studied (6), this is the first study to evaluate SHetA2 effects on hsc70. The observation that SHetA2 disrupts hsc70/AIF complexes suggests that, in addition to SHetA2 disruption of mortalin complexes, its disruption of hsc70 and potentially Grp78 complexes could contribute to the mechanism of SHetA2 cytotoxicity. Detailed studies are needed to determine which disruptions contribute to the cytotoxicity, which may counteract the cytotoxicity and/or which may have no effect. This knowledge would be valuable for the development of improved SHetA2 analogs with refined binding profiles to maximize the cytotoxicity while avoiding increased non-specific toxicity. The observation that elevated hsc70 is associated with reduced cervical cancer patient survivability suggest that hsc70 is a relevant target for cervical cancer drug development that warrants further study.

## Materials and methods

### Cell lines, culture conditions, and chemicals

Authenticated HPV-positive SiHa (RRID : CVCL_0032), Ca Ski (RRID : CVCL_1100), ME-180 (RRID : CVCL_1401), C-4-II (RRID : CVCL_1095), and HPV-negative C-33 A (RRID : CVCL_1094) cell lines were purchased from American Type Culture Collection (ATCC, Manassas, VA, USA) within the last three years and used within 20 passages when verified as mycoplasma-free. All the cell lines were authenticated by ATCC. SiHa, Ca Ski and C-33 A cancer cells were grown in RPMI 1640 medium (R8758, Sigma-Aldrich, Saint Louis, MO, USA), C-4 II cells were grown in Waymouth’s medium (# 11220035, Fisher Scientific) and ME-180 cells were grown in McCoy’s 5A Medium (#30-2007, ATCC). All the media were supplemented with 10% fetal bovine serum (Serum Source International- FBS17712) and 1% Antibiotic-Antimycotic Solution (ABL02- 100X, Caisson Labs, Smithfield, UT, USA). All experiments were performed with mycoplasma-free cells.

SHetA2 compound synthesized by K. Darrell Berlin at Oklahoma State University as published previously for product 15c in (46) was used in tissue culture studies. For all the cell culture studies, a 10 mM stock solution of SHetA2 was dissolved in dimethylsulfoxide (DMSO, Neta Scientific, Hainesport, NJ, USA). Untreated control cultures were treated with the same final volume of DMSO administered to the treated cultures. For *in vivo* studies, SHetA2 synthesized in bulk by the US National Cancer Institute RAID Program was suspended in 30% Kolliphor HS 15 (SigmaAldrich, Merck, Darmstadt, Germany) in water for use in the animal model. Untreated control animals were gavaged with the same volume of 30% Kolliphor HS 15 given to the treated animals.

### Mitochondrial membrane potential assay

Alterations in the MMP were determined using the MMP assay kit (ab113850; Abcam, Cambridge, MA, USA), which utilizes the JC-1 fluorescent dye (5′,6,′-tetrachloro-1,1′,3,3′ tetraethylbenzimidazolyl carbocyanine iodide) as described by the manufacturer’s instruction. In brief, cervical cancer cells (1.5 × 10^4^ cells/well) were seeded onto 96-well black plates (#NC1463153, Perkin Elmer, Waltham, MA, USA). Following SHetA2 treatment over a range of times, cells were probed with JC-1 (20 µM) for 20 min at 37°C and washed twice. Fluorescence intensity was measured using a microplate reader for aggregates (excitation at 535 nm and emission at 590 nm) and monomer (excitation at and 475 nm and emission at 530 nm). Change in MMP (Δψ_m._) was calculated as the ratio of J-aggregates to J-monomers. Depolarized mitochondria are indicated by decreased (Δψ_m._).

### ATP assay

CellTiter-Glo 2.0 Luminescent Cell Viability Assay (#G9241, Promega, Madison, WI, USA) was used and manufacturer’s instructions were followed to measure total cellular ATP levels. Briefly, human cervical cancer cells were seeded into 96-well plates at a density of 1 × 10^4^ and allowed to attach overnight followed by SHetA2 treatment for desired time. Afterwards, cells were incubated with CellTiter-Glo reagent and lysed. The luminescence signal was measured using a SYNERGY H1 microplate reader (BioTek, Winooski, VT, USA).

### Mitochondrial ROS

Mitochondrial ROS were measured using MitoSOX (Invitrogen) staining. Cervical cells were treated with SHetA2 for 24 hours followed by incubation with MitoSox Red (2.5 μM) for 30 min at 37°C. Data were acquired with a FACS Calibur (BD Biosciences) and analyzed with Flow Jo analytical software.

### Confocal microscopy for mitochondrial morphology

To assess mitochondrial morphology, cells were stained with MitoTracker™ Green FM (M7514, Invitrogen, USA). Approximately 6-8 × 10^3^ cells were plated on chambered slides and treated with SHetA2 for 24 hours. Then, the cells were stained with 100 nM Mitotracker**™** Green FM **(**Invitrogen) for 30 min at 37°C; after three times washing with PBS, the cells were incubated with 0.1 μg/mL Hoechst. Again, cells were washed twice with PBS and warm medium was administered. Live cell images were acquired with a 63X objective using a Zeiss Axio Observer. Z1 (Göttingen, Germany).

### Analysis of mitochondrial mass

To assess the mitochondrial mass, approximately 6-8 × 10^3^ cells were plated on 96-well black plates (#NC1463153, Perkin Elmer, Waltham, MA, USA) and treated with SHetA2 or vehicle for 24 hours. Then, the cells were stained with Mitotracker™ Green FM (100 nM, Invitrogen) for 30 min at 37°C; after three times washing with PBS, the cells were incubated with 0.1 μg/mL Hoechst. Then cells were washed twice with PBS and images were captured with the Operetta High Content Imaging System (Perkin Elmer, Waltham, MA, USA). Quantification of staining was done using Harmony Software (Perkin Elmer) to determine mitochondrial mass by the intensity of Mito Tracker green. Numbers of cells were determined by the number of nuclei as detected with Hoechst 33342 staining. The mitochondrial mass measurements were normalized to number of cells.

### Transmission electron microscopy

SiHa cells grown in tissue culture in the presence or absence of 10 µM SHetA2, or solvent only, for 4- or 8-hours were fixed overnight at 4°C in 4% paraformaldehyde/2% glutaraldehyde in 0.1M Cacodylate buffer, then post-fixed with 1% OsO4 for one hour. Sample dehydration was performed using graded ethanol and propylene oxide prior to infiltration with Epon/Araldite resin, embedding, and polymerization. Ultrathin (~90nm) sections were cut on a Leica Ultramicrotome and placed on 300 mesh copper grids. Grids were stained with lead for contrast using a standard protocol (Sat. Uranyl Acetate for 30 minutes) and Lead Citrate for 15 minutes). Sections on the grids were visualized using a Hitachi H-7600 Transmission Electron Microscope (at 80 kV).

Xenograft tissues generated as described in xenograft study were processed similarly except for a addition of dehydration step with acetone and propylene oxide.

### Western blot analysis

Whole-cell protein extracts of treated and control cervical cancer cells were isolated using the M-PER Mammalian Protein Extraction Reagent (#78501Thermofisher scientific, Waltham, MA, USA) supplemented with 1% phosphatase inhibitor cocktail (#4906845001 Sigma-Aldrich) and 1% protease inhibitor cocktail (#5892791001, Sigma-Aldrich). Mitochondria and cytoplasm-enriched fractions were isolated using Mitochondria/Cytosol Fractionation Kit (# ab65320, Abcam) according to the manufacturer’s protocol. Protein concentrations were determined using the BCA assay reagent (#23225 Thermofisher scientific, Waltham, MA, USA). Equal amounts of protein lysates (30µg protein) were electrophoresed into a 12% sodium dodecyl sulfate-polyacrylamide gel electrophoresis (SDS-PAGE) gel (Bio Rad, California, USA), and then transferred to polyvinylidene difluoride (PVDF) membrane (Bio Rad, California, USA) using a Trans-Blot^®^ Turbo™ Transfer System. The membranes were blocked with Tris Buffered Saline with 0.1% v/v Tween-20 (TBST) containing 10% dry skim milk for 1 hour at room temperature. Subsequently, membranes were probed with the primary antibodies overnight at 4°C. Primary antibodies; DRP1 (#5391), OPA1 (#80471), MFN1 (#14739), MFN2 (#11925), LC-3A/B (#12741), Cleaved Caspase-3 (#9664), AIF (#4642), γH2AX (#2577), Pink1 (#6946), Parkin (2132), VDAC (#4661), LC3A/B (#12741), P62/SQSTM1, GAPDH (#5174), α-Tubulin (#2125), Cyclophilin B (#43603) were purchased from Cell Signaling Technology. AIF (MA5-15880) and phospho (Ser65) Parkin (orb312554) antibodies were purchased from Thermo Fisher Scientific and Biorbyt, respectively. The primary antibody to hsc70 [EP1531Y] (ab51052) was purchased from Abcam. After three times washing with TBST, the membranes were incubated with horse radish peroxidase (HRP) conjugated anti-rabbit IgG (1:5000) or anti-mouse IgG (1:6000) for 45 min at room temperature followed by additional washing. After washing, protein bands were detected using the Enhanced Chemiluminescence (Clarity™ Western ECL Substrate #1705060S, BioRad, Hercules, CA, USA) and the Chemidoc Touch Imaging System (Bio Rad, Hercules, CA, USA) according to the manufacturer’s instructions. GAPDH/cyclophilin B or α tubulin was used as loading controls. Densitometric analysis was performed using Image Lab software (BioRad, Hercules, CA, USA).

### Cell viability Assay

Cell viability was measured using the 3-(4,5-dimethylthiazol-2-yl)-2, 5-diphenyl-tetrazolium bromide assay (MTT assay; #G4100, Promega Madison, WI, USA). Briefly, cells were harvested from culture dishes using 0.05% trypsin-0.02% EDTA, and cultured overnight in 96 well plates at densities of 4 000 cells/well. Then cells were treated with 0 - 10 µM of SHetA2 for 24- or 72-hours followed by addition of MTT solution (15µl). After a 2-hour incubation in the presence of MTT solution, solubilizing or STOP solution was added and the culture plate was incubated overnight. The optical density (OD) was measured at a wavelength of 570 & 620 nm using a BioTek Synergy H1 Micro Plate Reader and Gen5 2.09 Software. The average ODs of triplicate treatments were normalized to the average ODs of the cultures treated with DMSO solvent only. Prism 8 software (GraphPad, San Diego, CA, USA) was used to plot the normalized ODs against the SHetA2 concentrations used and to derive the half maximal inhibitory concentrations (IC_50_s/potencies), and efficacies (maximal percent growth inhibition activities) using nonlinear regression. For each cell line, experiments were performed in triplicate and repeated 2-3 times with consistent results.

### Caspase-3 activity assay

Caspase-3 activity was measured using Caspase 3, Caspase 8, and Caspase 9 Multiplex Activity Assay Kit (Fluorometric#ab219915, Abcam, Cambridge, MA, USA). Briefly, 1.5 X 10^4^ cells were seeded in 96-well black plate for overnight and treated with SHetA2 (10 μM) for 48 hours. Then Caspase 3 substrate was added to each well and the plate was incubated at room temperature for 60 minutes in dark. The fluorescence intensity was measured with using a Bio-tek Synergy H1 Micro Plate Reader and Gen5 Software at *E*x/*E*m of 535/620 nm for caspase-3.

### Immunofluorescence microscopy

Expression and/or localization of AIF, γH2AX and PINK1 were investigated by immunofluorescence microscopy. In brief, approximately 6-8000 cervical cancer cells were seeded on 8-chambered slides, and treated with SHetA2 or vehicle for 24 hours. Then the cells were fixed with 4% paraformaldehyde and permeabilized with 0.1% TritonX‐100 in PBS. Following blocking with 4% BSA in PBS, cells were incubated with and MitoTracker™ Red CMXRos (#M7512, Thermo Fisher Scientific) for 45 minutes and then by primary antibodies for AIF (#MA5-15880, Thermo Fisher Scientific) or γH2AX or PINK1 at 1:100 dilutions in 1% BSA‐PBS for overnight. After washing, cells were stained with Alexa Fluor 488‐labeled secondary antibody for 1 hour. DAPI (blue) was used to stain the nucleus. Cell images were acquired with a 63X objective using a Zeiss Axio Observer Z1 (Göttingen, Germany). For mitophagy detection, cervical cancer cells treated with SHetA2 or DMSO were stained with LysoTracker™ Deep Red (#L12492, Thermo Fisher Scientific) and MitoTracker™ Green FM, and live-cell imaging was performed with a 63X objective using a Zeiss Axio Observer Z1 (Göttingen).

### Co-immunoprecipitation assay

For the co-immunoprecipitation assay, cervical cancer cells were treated with SHetA2 or vehicle for 24 hours and protein isolates were collected with M-PER. Approximately 500 μg of protein lysate was incubated with agarose beads (already coupled with 10 µg of hsc70 or AIF antibodies per manufacturer protocol of PierceTM Crosslink IP kit, ThermoFisher Scientific # 26147) overnight at 4°C. Then beads were washed with buffer provided in the kit and the immuno-precipitated complexes were collected, re-suspended in sample buffer and heated for 5 min at 95°C. The co-immunoprecipitation of client proteins was detected by Western blot analysis using equal volumes of immuno-precipitated proteins.

### Xenograft study

The animal study was approved by the University of Oklahoma Health Sciences Center Institutional Animal Care and Use Committee (IACUC Protocol #19-009-CHI). After an acclimation period of two weeks, female athymic Hsd : Athymic Nude-*Foxn1nu* mice (four-week old, ENVIGO, Alice, TX, USA) were subcutaneously injected with 5 × 10^6^ Ca Ski cells suspended in 1× phosphate buffered saline (PBS). Tumor sizes were measured thrice per week with calipers. Once the tumors achieved ~200 mm^3^ average tumor volume ([width^2^ × length]/2), mice were randomized into 3 animals/treatment group based on tumor volume so that there were no significant differences between the groups (ANOVA, *p* > 0.05) and treatment was initiated. SHetA2 was orally administered daily for 21 days at doses of 30 mg/kg and 60 mg/kg while the control group was gavaged with placebo (30% Kolliphor HS 15 in water). At the end of the study period, animals were sacrificed. Tumors were collected and weighed at the time of necropsy. Tumors in the 30 mg/kg/day dose group were fixed for transmission electron microscopy analysis. Tumors in the 60 mg/kg/day dose group were fixed for immunohistochemical staining.

### Statistical analysis

All the experiments were independently repeated at least twice or thrice and in triplicate wherever applicable. Data are expressed as mean ± standard deviation (SD) for experimental replicates and standard deviation of the mean (SEM) for biological replicates. The *t*‐test and ANOVA were used to make comparisons between two groups or multiple groups, respectively. In situations where the data was not normally distributed, the Mann-Whitney test or Kursal-Wallis test, were used for two groups or multiple groups, respectively P <0.05 was considered statistically significant. Statistical analyses were done using GraphPad Prism 8 or 9 Software (GraphPad Software Inc., La Jolla, CA, USA).

## Data availability statement

The original contributions presented in the study are included in the article/**Supplementary Material**. Further inquiries can be directed to the corresponding author.

## Ethics statement

The animal study was reviewed and approved by University of Oklahoma Health Sciences Center Institutional Animal Care and Use Committee.

## Author contributions

Conception and Design: RR and DB; Development of Methodology: RR, VC; Acquisition and analysis of data; RR for all, VC for mitochondrial network analysis and confocal imaging, AK for xenograft tumor growth, RZ for quantification of immunohistochemical staining of tumors, DB for bioinformatics; Interpretation of data: RR, VC and DB; Writing of manuscript: RR and DB; Review of manuscript: AK, VC, RR, DB; Administrative, technical or material support; RR and DB; Study Supervision: RR and DB. All authors read and approved the final manuscript.

## Funding

This research was funded by the National Cancer Institute grant R01CA200126. Research reported in this publication was supported by National Cancer Institute (NCI) grant R01CA200126 (DB) and in part by the NCI Cancer Center Support Grant P30CA225520 awarded to the University of Oklahoma Stephenson Cancer Center and used the Molecular Biology and Cytometry Research and the Biospecimen and Tissue Pathology Shared Resources.

## Acknowledgments

Transmission electron microscopy was performed by the Oklahoma Medical Research Foundation Imaging Core Facility. Illustration created with BioRender.com.

## Conflict of interest

The authors declare that the research was conducted in the absence of any commercial or financial relationships that could be construed as a potential conflict of interest.

## Publisher’s note

All claims expressed in this article are solely those of the authors and do not necessarily represent those of their affiliated organizations, or those of the publisher, the editors and the reviewers. Any product that may be evaluated in this article, or claim that may be made by its manufacturer, is not guaranteed or endorsed by the publisher.

## Author disclaimer

The content is solely the responsibility of the authors and does not necessarily represent the official views of the National Institutes of Health.

## Supplementary material

Supplementary Figure 1 SHetA2 induces mitochondrial ROS in cervical cancer cells: **(A, B)**, ME-180 and C-4-II cervical cancer cells were treated with 10 µM SHetA2 or vehicle for 24 hours followed by MitoSOX staining and analyzed by flow cytometry. Representative staining histogram depicting % MitoSOX positive cells are shown (left panel). Bar graphs (right panel) show the mean ±SD of three independent experiments and a t-test was used for statistical analysis. **(C)**, C-33 A, Ca Ski and SiHa cervical cancer cells treated with 10 µM SHetA2 or vehicle for 24 hours were stained with MitoTracker™ Green and Hoechst. The fluorescence was imaged and analyzed by using the Operetta®High Content Imaging System. ** p ≤ 0.01, **** p ≤ 0.0001 when compared with respective control.

Supplementary Figure 2 SHetA2 induces mitophagy in cervical cancer cells: Mitophagy induction was demonstrated by confocal imaging of cervical cancer cells treated with SHetA2 (10 µM) for 24 hours and stained with Pink1 (green) mitotracker red (red) and DAPI (blue). Colocalized (Yellow) spots for Mitoview and Pink1 were counted using ImageJ software.
