# Supplementary figures and images for: Distinct mechanism of cervical cancer cell death caused by the investigational new drug SHetA2
Source: Front Oncol. 2022 Sep 20;12:958536. doi: 10.3389/fonc.2022.958536 (PMC9531157; doi:10.3389/fonc.2022.958536)

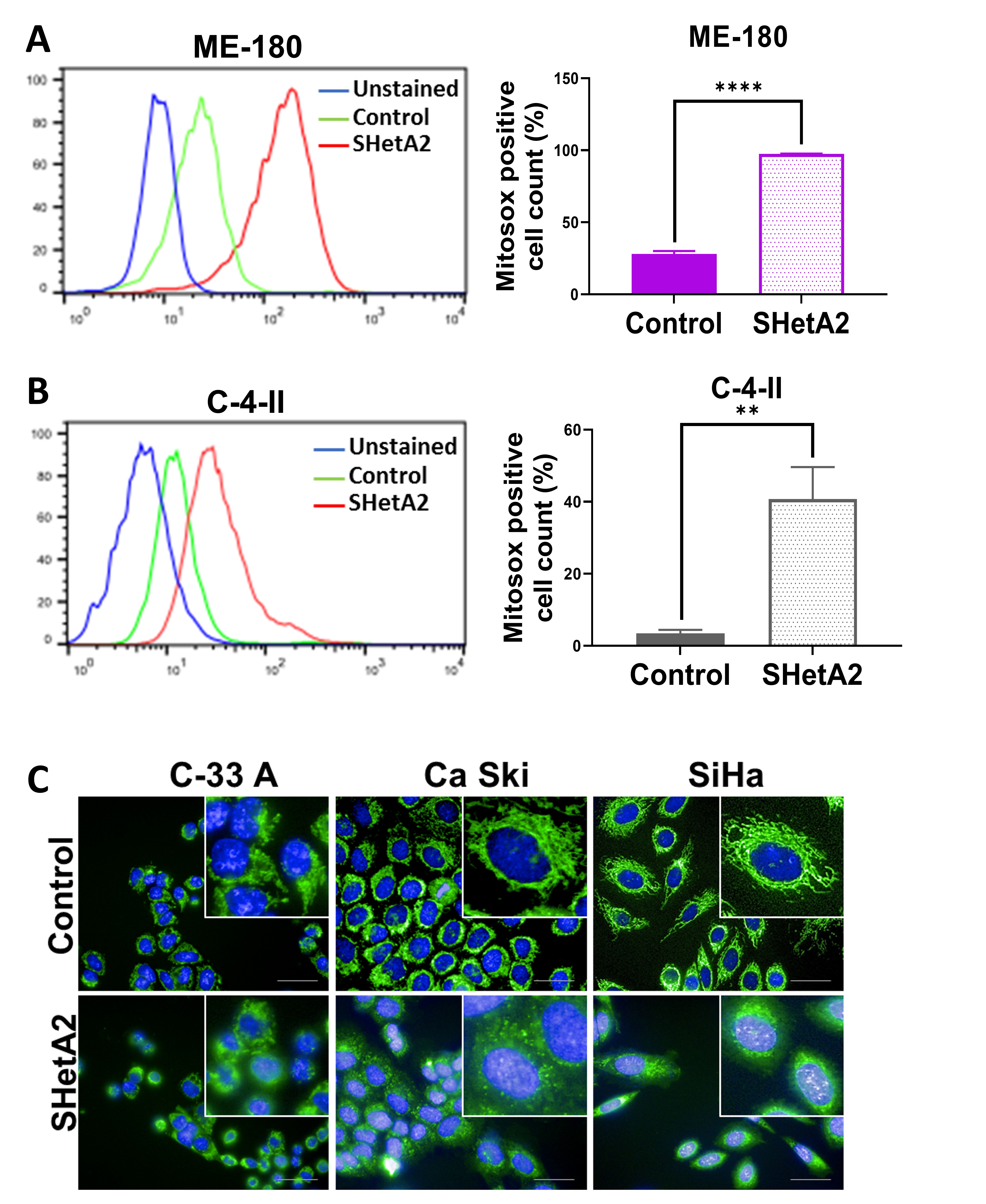

Supplement: Supplementary Figure 1 — SHetA2 induces mitochondrial ROS in cervical cancer cells: (A, B), ME-180 and C-4-II cervical cancer cells were treated with 10 µM SHetA2 or vehicle for 24 hours followed by MitoSOX staining and analyzed by flow cytometry. Representative staining histogram depicting % MitoSOX positive cells are shown (left panel). Bar graphs (right panel) show the mean ±SD of three independent experiments and a t-test was used for statistical analysis. (C), C-33 A, Ca Ski and SiHa cervical cancer cells treated with 10 µM SHetA2 or vehicle for 24 hours were stained with MitoTracker™ Green and Hoechst. The fluorescence was imaged and analyzed by using the Operetta®High Content Imaging System. ** p ≤ 0.01, **** p ≤ 0.0001 when compared with respective control. [file Image_1.jpg]

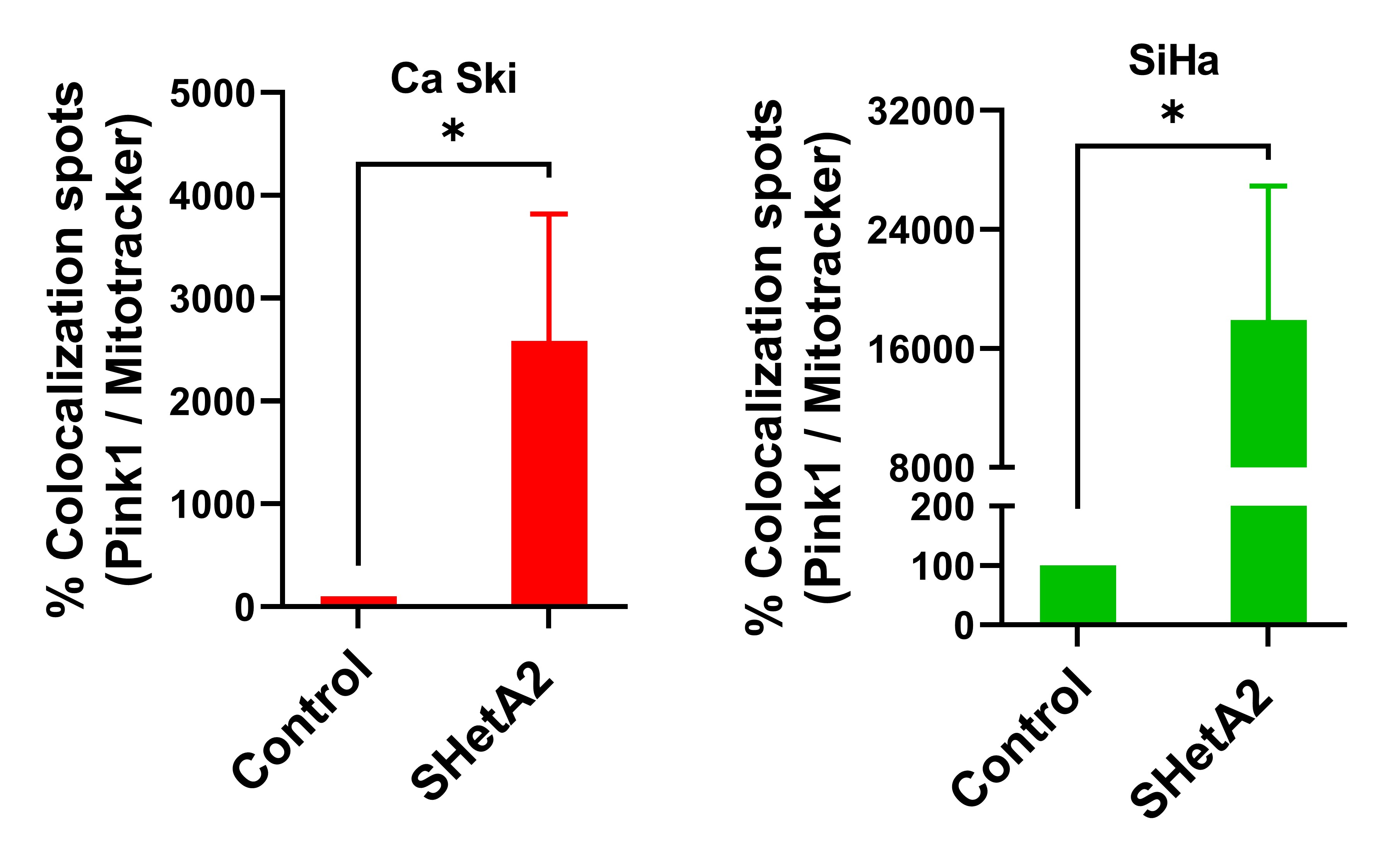

Supplement: Supplementary Figure 2 — SHetA2 induces mitophagy in cervical cancer cells: Mitophagy induction was demonstrated by confocal imaging of cervical cancer cells treated with SHetA2 (10 µM) for 24 hours and stained with Pink1 (green) mitotracker red (red) and DAPI (blue). Colocalized (Yellow) spots for Mitoview and Pink1 were counted using ImageJ software. [file Image_2.jpg]
